# Development of the Parent–Child Communication Quality Scale from the Perspective of Children’s Psychological Needs

**DOI:** 10.3390/bs14100933

**Published:** 2024-10-11

**Authors:** Wenhui Lyu, Xiaohui Shi, Zhiheng Xiong, Yujie Mu

**Affiliations:** 1 School of Educational Science, Nanjing Normal University of Special Education, Nanjing 210038, China; 210028@njts.edu.cn; 2 School of Special Education, Nanjing Normal University of Special Education, Nanjing 210038, China; 3 School of Humanities, Southeast University, Nanjing 211189, China

**Keywords:** parent–child communication, self psychology, developmental psychological needs, psychometric study

## Abstract

Parent–child communication plays a crucial role in children’s healthy growth. Nevertheless, there is currently a paucity of instruments designed to measure the quality of parent–child communication from a psychological perspective. Accordingly, based on the self psychology theory, this study has developed the Parent–Child Communication Quality Scale (PCCQS) to assess the quality of parent–child communication in terms of the extent to which children’s psychological needs are met. A total of 1095 urban children (50.9% girls, *M* = 9.92, *SD* = 1.15) aged 8 to 12 years in eastern China were surveyed in this study. The measurement structure of the PCCQS was examined using an exploratory factor analysis (EFA) and a confirmatory factor analysis (CFA). The results of the CFA supported the second-order, four-factor structure of the PCCQS, with the 15-item PCCQS consisting of four factors (i.e., mirroring, idealization, empathy, and appropriate response). In conclusion, the PCCQS has good construct and criterion validity, as well as high internal consistency and split-half reliability. The scale is therefore a valuable tool for assessing the quality of parent–child communication.

## 1. Introduction

Parent–child communication can be defined as the process of exchanging information between parents and children through verbal and non-verbal forms, with the aim of expressing thoughts, opinions, and emotional attitudes. As the earliest emergence of frequent and long-lasting interpersonal interactions in children’s lives, it has a profound impact on children’s development. In terms of children’s psychological development, parent–child communication has been confirmed to affect children’s attachment [1], self-esteem [2], psychological resilience [3], and well-being [4]. A systematic review of 37 articles revealed a negative association between parent–child communication and adolescent mental health problems (e.g., depression, anxiety, psychosis, and suicidal ideations) [5]. A longitudinal study demonstrated that a lack of parent–child communication at age 10 was a significant predictor of depression 20 years later [6]. Concerning children’s behavioral expression, parent–child communication quality has been found to have a positive association with children’s academic performance [7], academic engagement [8], interpersonal adaptation [9], and prosocial behavior [10]. Simultaneously, it has also been confirmed to be negatively associated with addictive behaviors [11] and behavioral problems [12]. In addition, a two-year longitudinal study indicated that failure in parent–child communication was a contributing factor to the adverse effects of digital violence in games during adolescent development, which was closely associated with direct aggressive behavior [13]. A cross-sectional study conducted on 2751 high school students in China revealed a negative correlation between the perceived quality of parent–adolescent communication and pathological Internet use [14].

The current perspectives for measuring parent–child communication are primarily concentrated on parent and child self-reporting, with limited studies utilizing third-party observation scales [15,16]. Some studies suggested that the results of parent–child communication, as measured from the three aforementioned perspectives, were relatively independent [15,17]. For instance, the descriptions provided by adolescents of their mothers’ communication are typically less correlated with their mothers’ self-reports [18]. The discrepancy in results measured by the disparate parent–child communication informants does not imply that one perspective is inherently more accurate than another. In clinical child studies, it can be frequently observed that different informants assign varying ratings to children’s social, emotional, or behavioral problems. These discrepancies are primarily attributed to differences in the attributions of children’s behavioral issues between children and their parents and teachers, as well as differentiation in their recall and attention to information [19]. Furthermore, in daily life, it can be observed that children and parents often exhibit disparate behaviors and concerns in parent–child communication. This difference has been interpreted by anthropologists as the generation gap. Accordingly, to accurately assess parent–child communication, it is crucial to select an appropriate measurement perspective that aligns with this study’s objectives.

The present study is intended to measure the quality of parent–child communication from the perspective of the children’s psychological needs. It can be argued that the children’s communication experiences are more closely related to child development than the parents’ communication experiences, which may help professionals design effective child-centered interventions. Two principal instruments are currently employed for the measurement of parent–child communication from the child’s perspective. The Parent–Adolescent Communication Scale (PACS), devised by Barnes and Olson in 1982, is the most widely used instrument for the assessment of the quality of parent–child communication. It was employed as a measure of parent–child communication in 86% and 75% of the studies included in two systematic reviews of parent–child communication [5,20]. The PACS consists of two subscales, including openness in family communication (Open Family Communication Scale), and problems in family communication (Problems in Family Communication Scale). It is available in two separate forms, which were given to samples of parents and adolescents [21]. Although PACS has a significant impact on the measurement of parent–child communication quality and the development of subsequent scales, it lacks a clear theoretical foundation, and the two dimensions are merely a list of communication manifestations. The second, more widely used instrument is the Revised Family Communication Pattern (RFCP) by Ritchie and Fitzpatrick [22], which is divided into two dimensions(i.e., socio-orientation and concept-orientation) to judge the type of parent–child communication. The two dimensions of the RFCP reflect the characteristics of parent–child communication; however, it is not a direct measure of the quality of parent–child communication. Furthermore, there are some other parent–child communication measurement tools that are occasionally employed. These are often developed based on clinical experience and lack theoretical support and specific construct validity. For example, the children’s version of the Parent–Child Communication Scale (PCCS McCarty) employs a 10-item scale to assess children’s perceptions of their primary caregiver’s openness to communication [23], while the Parent–Adolescent Communication Inventory (PACI) investigates adolescents’ perceptions using 36 items to measure healthy parent–child communication and disordered parent–child communication [24]. However, PACI lacks a clear dimensional division, with items pertaining to fathers, mothers, and both parents intermingled.

Furthermore, there are two principal measurement instruments in China that can measure the quality of parent–child communication from the child’s perspective. The Adolescent Parent–Child Communication Questionnaire, developed by Yang and Zou, is divided into four dimensions: open expression and communication, listening and responding, disagreement and conflict, and resolution and comprehensibility [25]. This instrument explores the characteristics of adolescent parent–child communication at the phenomenological level. Chi proposed a three-level (i.e., component, relationship, and system) model of parent–child communication, and accordingly developed three subscales of parent–child communication skills, quality, and systems [26]. Among these, the quality subscale examines the quality of dyadic communication occurring between father and child or mother and child from the two dimensions of relational and problem orientation [26]. The scale focuses on a comprehensive and objective assessment of both parent–child communication ability and function, but does not measure the quality of parent–child communication from the perspective of meeting children’s psychological needs.

Based on the aforementioned review of the parent–child communication scale, it is observable that the existing measurement tools for parent–child communication from the perspective of children primarily assess the function, mode, characteristics, and communication ability of the parent–child communication. There is no consensus within the academic community regarding how to measure the quality of parent–child communication from the perspective of children’s psychological needs. The existing measurement tools frequently lack theoretical foundations and exhibit a simple division of dimensions, potentially neglecting some crucial aspects of parent–child communication. This study aims to provide a concise and effective tool for relevant professionals to assess the quality of parent–child communication and lay a measurement foundation for further exploring the mechanisms underlying the impact of parent–child communication on children’s psychological development.

## 2. Materials and Methods

This study defined parent–child communication quality as the extent to which parents meet the psychological needs of children’s development through verbal and non-verbal information exchange. Based on the self psychology theory, a Parent–Child Communication Quality Scale (PCCQS) from the perspective of children’s psychological needs was developed.

### 2.1. Research Design

In accordance with the fundamental principles of scale development and validation, this study was conducted in three phases. Firstly, a second-order, four-factor measurement model of parent–child communication was constructed based on the self psychology theory to clarify the meaning and relationship of the four factors. Secondly, initial items for the scale were developed through open interviews, which were then reviewed by experts and pilot-tested among primary school students. After modifying some items based on feedback from experts and students, a scale suitable for a large-scale measurement was finalized. Finally, the scale was administered to primary school students to assess its quality, and a formal scale was developed based on the results.

### 2.2. Theoretical Conception of the Scale

The self psychology theory is one of the psychoanalytical theories developed by Heinz Kohut in the 1970s, based on the clinical practice of counselling. This theory explores the development of the individual at the level of the human subject’s experience, and refers to the basic psychological needs of human development as selfobject needs. These needs should be satisfied to facilitate an optimal development throughout the individual’s lifespan, whether in childhood, adolescence, or adulthood, or in the context of a psychotherapeutic relationship. Kohut posits that individuals rely on the responses of others to meet their selfobject needs and identifies three specific clusters of developmental needs: mirroring, idealization, and twinship [27].

**Figure 1:**
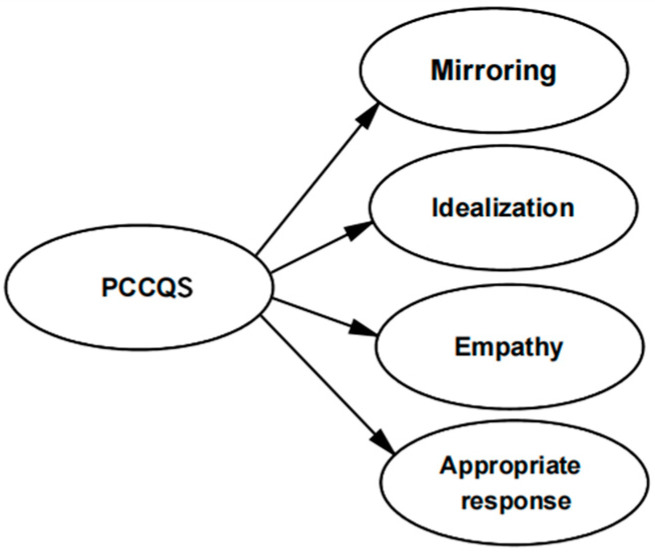
The second-order, four-factor measurement model of the Parent–Child Communication Quality Scale (PCCQS).

This study focused on the quality of parent–child communication experienced by the children. The twinship needs often arise among peers. Therefore, in the design of the scale, the functions of parents in satisfying children’s need for mirroring and idealization in parent–child communication were mainly considered. Based on this, a second-order measurement model containing the four factors of mirroring, idealization, empathy, and appropriate response was constructed (see Figure 1).

Factor 1, mirroring, represents the selfobject function of parents in fulfilling children’s need for mirroring during communication. It signifies the extent to which children perceive their parents as supportive in building and maintaining their self-esteem and self-confidence. Factor 2, idealization, represents the selfobject function of parents in meeting children’s need for idealization in communication. It specifically refers to the extent to which children in parent–child communication feel linked to their parents in a way that meets their own needs for a sense of security, calmness, and the experience of comfort. Factor 3 is empathy, which can be defined as the ability of parents to gather information to respond in an appropriate manner to the child’s selfobject needs. This factor refers to the capacity of parents to empathically engage with their children, without subjectively judging their inner experiences. This is primarily evidenced by the parents’ ability to accurately discern their children’s emotional states and to attentively listen to and comprehend their children’s thoughts and feelings during parent–child interactions. Factor 4, appropriate response, pertains to the parent’s capacity to respond to the child’s driving demands with a non-hostile and non-tempting emotional demeanor. A high level of appropriate response enables the child to experience optimal frustration while avoiding traumatic frustration experiences. In this context, the term ‘drive’ is used to describe the psychic energy associated with the subconscious system and the fulfilment of desires. If the parent’s response to the child’s demand is not traumatic, the child will be able to satisfy their own desires in a calm and loving manner, rather than in a counterattacking manner [28]. Self psychology emphasizes that optimal frustration can promote individual psychological growth. In parent–child communication, when children experience external losses or disappointments safely without overwhelming their emotional capacity, these experiences do not result in trauma. Instead, children can achieve psychological growth by exposure to manageable challenges that they can overcome.

### 2.3. Scale Development

The four factors of mirroring, idealization, empathy, and appropriate response were used as a framework for the design of a semi-structured interview outline to assess the quality of children’s parent–child communication. A sample of 10 primary school students was selected for semi-structured interviews to initially assess the plausibility of the theoretical construction and to collect relevant information for the development of the scale. Following an analysis of the interview data, the four factors were confirmed as plausible, and the initial scale was developed. Six experts in the field of self psychology were invited to assess the extent to which the scale items reflected the relevant dimensions, and modifications and adjustments were made to the scale based on their feedback. Subsequently, 100 primary school students in grades 3 to 6 were selected for the pre-test, and then the items that were ambiguous or obscure were refined according to the results of the pre-test. Finally, a 16-item scale for the children’s self-reports were completed, comprising four factors: mirroring, idealization, empathy, and appropriate response. The scale was scored using a Likert 5-point scale, ranging from 1 = strongly disagree to 5 = strongly agree, with a higher total score indicating a higher quality of parent–child communication. Given the potential differences between father–child communication and mother–child communication, we divided the PCCQS into a father’s version and a mother’s version, which were designed to separately measure the degree to which fathers and mothers satisfy children’s developmental psychological needs during communication. The items in both versions were expressed in exactly the same way.

### 2.4. Participants and Procedure

This study recruited 1095 urban primary school students (50.9% girls) from nine primary schools in eastern China through convenience sampling. The participants were aged between 8 and 12 years old (*M* = 9.92, *SD* = 1.15), with 179 (16.3%) in the third grade, 417 (38.1%) in the fourth grade, 222 (20.3%) in the fifth grade, and 277 (25.3%) in the sixth grade. The survey was conducted in a classroom setting with the assistance of trained testers, who distributed paper forms, read standardized instructions, and responded to questions. This study was approved by the Ethics Committee of Nanjing Normal University of Special Education (protocol code: NJTS20240319022).

The data from the officially administered sample were randomly divided into two groups, designated as A and B. Group A, comprising 545 participants, was employed for an item analysis and exploratory factor analysis (EFA), while Group B, comprising 550 participants, was utilized for a confirmatory factor analysis (CFA).

### 2.5. Validation Instruments

In this study, the Parent–Adolescent Communication Scale (PACS) (adolescent version), developed by Barnes and Olson, was employed as the instrument for assessing the validity of the PCCQS. The PACS comprises two dimensions, namely openness in family communication (OFC) and problems in family communication (PFC), with 10 items in each dimension. It is scored on a 5-point Likert scale, with higher scores indicating greater levels of parent–child communication. The PFC dimension is reverse scored, with higher scores indicating fewer parent–child communication problems. In this study, the PACS exhibited good internal consistency (α = 0.73).

### 2.6. Data Analysis

The data were processed using the SPSS 25.0 and Mplus 8.0. First, an item analysis was conducted with the SPSS 25.0 on Group A, including independent samples *t*-tests and Pearson’s correlation analyses employed to assess item discrimination and the degree of linear correlation between the items and the total scale scores, respectively. Second, we used the SPSS 25.0 to conduct an EFA on Group A to initially explore the construct validity of the scale; subsequently, the Mplus 8.0 was used to examine the CFA of Group B to verify whether the construct validity of the scale met our expectations. Finally, reliability tests were conducted on the full sample using the SPSS 25.0, including internal consistency reliabilities and split-half reliabilities, to assess the consistency and reliability of the scale. Finally, we used the PACS as the criterion, and tested the criterion validity of the full sample with the SPSS 25.0 to verify the validity of the whole scale.

## 3. Results

### 3.1. Item Analysis

The Parent–Child Communication Quality Scale was analyzed using the critical ratio (C.R.) value and correlation analysis. Group A was sorted according to the total score of the father’s version, and independent samples t-tests were conducted for the high subgroup with the top 27% of scores (total score ≥ 76) and the low subgroup with the bottom 27% (total score ≤ 58). The results demonstrated that the C.R. value for all items of the father’s version ranged from 17.772 to 26.180 (*p* < 0.001), indicating a statistically significant difference in the scores of all items in the high and low subgroups of the father’s version. The Pearson’s correlation coefficients of the items of the father’s version with the total score ranged from 0.71 to 0.86 (*p* < 0.01), indicating a significant positive correlation between the items and the total score. Then, Group A was sorted according to the total score of the mother’s version, and an independent samples *t*-test was performed on the high subgroup of the top 27% of the scores (total score ≥ 78) and the low subgroup of the bottom 27% (total score ≤ 63). The results demonstrated that the C.R. value for all items of the mother’s version ranged from 13.199 to 26.805 (*p* < 0.001), indicating all items in the high and low groups were significantly different. The Pearson’s correlation coefficients of the items of the mother’s version with the total score ranged from 0.68 to 0.85 (*p* < 0.001), indicating a significant positive correlation between the items and the total score. The results of this analysis demonstrated clear differentiation between the items, with no items being excluded.

### 3.2. Exploratory Factor Analysis (EFA)

The EFA was conducted on Group A comprising 545 participants. Factor extraction was performed using a principal component analysis, and an oblique rotation was carried out using the promax rotation. Items were excluded according to the following criteria: factor loadings below 0.4, commonality below 0.3, and the difference in loadings between two factors below 0.2.

The EFA results of the father’s version demonstrated KMO = 0.967, df = 120, and a Bartlett’s test of sphericity with *p* < 0.001. In contrast, the EFA results of the mother’s version exhibited KMO = 0.965, df = 120, and a Bartlett’s test of sphericity with *p* < 0.001. Both the results of the father’s and mother’s versions were suitable for the EFA.

In this study, two methods were employed to restrict the number of factors included in the EFA. The first method was to limit the extraction to 4 factors. The results of the analysis demonstrated that only one of the four factors extracted from the father’s version had an eigenvalue > 1, with a cumulative explained variance of 77.938%. In contrast, two of the four factors from the mother’s version had an eigenvalue > 1, with a cumulative explained variance of 75.294%. Item 16 “My parents will tell me gently when they can’t satisfy my needs” was excluded from the analysis due to the cross-loading of three factors in the father’s version, resulting in a 15-item formal scale. Then, the 15-item scale was subsequently analyzed by the EFA, and the results were presented in Table 1 for the father’s version and Table 2 for the mother’s version. The EFA results for the four factors of the PCCQS demonstrated that the attribution of the items was consistent with the theoretical assumptions. Specifically, items 5, 6, 7, and 8 were attributed to the Mirroring factor, items 1, 2, 3, and 4 to the Idealization factor, items 9, 10, 11, and 12 to the Empathy factor, and items 13, 14, and 15 to the Appropriate Response factor. The loadings of the items in the pattern matrix on the attributed factors ranged from 0.453 to 0.931, with no items cross-loading. The results indicate that the four factors exhibit a degree of differentiation. However, both the father and mother versions have two factors with eigenvalues of 0.6, which explains only approximately 4% of the variance. Thus, the EFA does not yet support the division of the four factors from the data. The reasonableness of the division of the four factors can be further verified by a CFA.

The second EFA method was constrained to the extraction of a single factor, based on the eigenvalues of the factors and the Scree Plot. The EFA results of the father’s version demonstrated that a single factor was responsible for 63.765% of the observed variance, with factor loadings ranging from 0.691 to 0.862 across the items (see Table 1). The EFA results of the mothers’ version demonstrated that a single factor could explain 60.853% of the variance, with factor loadings ranging from 0.660 to 0.849 across the items (see Table 2). The results of the aforementioned analyses indicate that the PCCQS can also be explained by a single latent factor, with all items exhibiting a closer relationship to this factor.

### 3.3. Confirmatory Factor Analysis (CFA)

A series of CFAs were conducted on the one-factor model, the first-order, four-factor model, and the second-order, four-factor model of the PCCQS for the 15 items, based on the 550 participants from Group B. A model fit is considered acceptable when χ^2^/df is less than five, the CFI and TLI are greater than 0.90, and the RMSEA and SRMR are less than 0.08 [29]. The results of these analyses demonstrated that the one-factor model for the father’s and mother’s versions exhibited the poorest fit and did not meet the established fit criteria [30], whereas the first-order, four-factor model and the second-order, four-factor model demonstrated a more satisfactory fit (see Table 3). The second-order, four-factor model exhibited slightly reduced values of χ^2^/df in comparison to the first-order, four-factor model, and the model was more simple and more in line with the theoretical conception of this scale. Furthermore, Marsh and Hocevar suggested that the ratio of the χ^2^ of the first-order model to that of the higher-order model could be employed as a target coefficient for determining the fitness of the model [31], with a target coefficient that is closer to one indicating the greater representativeness of the higher-order model [32]. The target coefficients for the father’s and mother’s versions in this study were 0.991 and 0.987, respectively, which is close to one, indicating that the second-order, four-factor model was a more representative model. The second-order, four-factor structure of the scale was validated through the CFA, which yielded standardized factor loadings for each item in the mother’s version ranging from 0.703 to 0.845 and dimensional loadings from 0.820 to 0.960. In contrast, the standardized factor loadings for each item in the father’s version ranged from 0.770 to 0.884, with dimensional loadings from 0.796 to 0.974.

### 3.4. Reliability Test

A reliability test was conducted on the formal test sample, comprising 1095 participants. The results of the reliability test, as presented in Table 4, demonstrated that the internal consistency and split-half reliability of the scale were satisfactory. The Cronbach’s alpha coefficients for each factor and the total scale of the father’s version exhibited a range from 0.859 to 0.960, while the split-half reliabilities demonstrated a range from 0.736 to 0.922. The Cronbach’s alpha coefficients for the mother’s version’s factors and total scale exhibited a range of 0.839 to 0.953, while the split-half reliabilities demonstrated a range of 0.706 to 0.927.

### 3.5. Test of Criterion Validity

In the present study, the Parent–Adolescent Communication Scale (PACS), developed by Barnes and Olson in 1982, was selected as the criterion for validity in the test of criterion validity. The results of the test demonstrated that the factors of the father’s version exhibited medium–high positive correlations with the subscale of openness in family communication (OFC) and the total scale of the PACS, with correlation coefficients ranging from 0.623 to 0.825 (see Table 5). The factors of the mother’s version also demonstrated medium–high positive correlations with the OFC and the total scale of the PACS, with correlation coefficients ranging from 0.586 to 0.804 (see Table 6). The factors of the father’s version exhibited only a low level of positive correlation with the subscale of problems in family communication (PFC) of the PACS (see Table 5), and the factors of the mother’s version also demonstrated only a low positive correlation with the PFC of the PACS (see Table 6). This is consistent with the degree of correlation between the two dimensions (i.e., OFC and PFC) of the PACS, which is related to the item content of the PFC of the PACS and the manner in which it is scored.

## 4. Discussion

Based on the self psychology theory, the purpose of this study was to measure the quality of parent–child communication from the perspective of the developmental psychological needs of the child. In the context of parent–child communication, parents, as primary caregivers in the child’s early life, play an important role in fulfilling the child’s selfobject needs. For instance, parents meet their children’s mirroring needs by acknowledging their positive qualities and accomplishments and fulfill their idealization needs through the creation of an idealized parental figure that the child identifies with. The satisfaction of these selfobject needs facilitates the child’s development of inner stability, security, and self-cohesion. When parents are able to respond to the child’s intrinsic needs, the child is able to gradually internalize the functions of self-regulation and become less dependent on external resources. This process is referred to as “transmuting internalization”, which bears resemblance to the ‘internal working model’ postulated by Bowlby in his attachment theory [33]. However, if parents fail to meet their children’s selfobject needs, the process of transmuting internalization may be blocked, resulting in pathological narcissism. The child may develop an avoidant personality disorder, characterized by a defensive avoidance of selfobject experiences and a denial of selfobject needs. The self psychology theory can elucidate the intrinsic relationship between parent–child communication and children’s psychological development. Its core concepts, including self, selfobject, selfobject needs, empathy, optimal frustration, and transmuting internalization, provide a theoretical framework for the measurement of parent–child communication.

The four factors, mirroring, idealization, empathy, and appropriate response, collectively constitute the measurement structure of parent–child communication quality. These factors not only assess the quality of parent–child communication in terms of its various dimensions, but also provide an overall evaluation of the quality of communication between parents and their children. The mirroring and idealization factors assess the extent to which parents are responsive to their children’s needs and desires. In contrast, the appropriate response factor measures the appropriate degree to which parents are responsive when they are unable to meet their children’s needs and desires. Empathy is an independent factor that exerts influence over the effects of the other three factors, and is therefore distinguished as a separate factor on the scale. The results of the CFA supported the rationality of the second-order, four-factor structure of the PCCQS, indicating that the aforementioned factors are effective in measuring the quality of parent–child communication.

From an experiential perspective, selfobject experiences among others tend to be compounded, which results in high correlations between the four factors. Idealization selfobject needs and mirroring selfobject needs are two distinct needs, clearly delineated by self psychology. However, they are closely intertwined at the experiential level. The fulfilment of idealization needs provides individuals with a sense of security and protection, which in turn enables them to present themselves more confidently. This, in turn, facilitates the satisfaction of mirroring needs. The fulfilment of mirroring needs enhances an individual’s self-esteem and sense of worth, making them more willing to seek and accept idealized support from others. In self psychology, optimal frustration is regarded as a necessary condition for the psychological growth of an individual, which encourages children to actively adjust their mental status to their environment in a way that is conducive to their development. It is only responses based on empathy that can facilitate growth without creating traumatic experiences for the child. The PCCQS employs the appropriate response factor to assess the quality of parents’ responses to their children’s needs and desires when they are unable to meet them. The factor comprises four items, one of which, “My parents will tell me gently when they can’t satisfy my needs” was excluded due to cross-loading with the mirroring, idealization, and appropriate response factors of the father’s version. It would be beneficial to further explore reasonable ways of expressing this item in the future. For example, excessive cross-loading may be avoided if it was modified to, “When my parents think my requests are unreasonable, they will reject me in a way that is acceptable to me”. Additionally, self psychology has also identified empathy as a mode of response. Being deeply understood is often a gratifying experience, relieving frustration and tension and promoting mental health [34], thus leading to a high correlation between the empathy factor and the mirroring and idealization factors.

During the scale development phase, the researchers conducted EFAs on multiple occasions using alternative data sets. Although the EFAs were constrained to extracting four factors, the item attributions of the factors were still consistent with the theoretical conception. However, in the majority of cases, only one factor with an eigenvalue > 1 could be identified. Furthermore, the combination of Scree Plot and parallel analyses also indicated that the extraction of a single factor was appropriate. This result may be attributed to the fact that the correlated components of the four factors are encompassed within the analysis of a common factor, which consequently leads to a higher variance being explained for this common factor. It has been suggested that when there is an a priori theory for the underlying factor structure of the data, a CFA should be considered as an alternative to an EFA [35]. The second-order, four-factor structure of the PCCQS is supported by a CFA, in addition to good criterion validity and reliability. Therefore, it can be used as an instrument to measure parent–child communication quality.

It should be noted that the present study still had several limitations. Due to the limitations of the research conditions, the participants in this study were restricted to primary school students, and the applicability of the PCCQS in children aged 8–12 can only be preliminarily verified. To ascertain its applicability in a group of adolescents aged 13–18 years old, the scale must be tested in a secondary school student group. Furthermore, the participants were all from China, and the findings must be tested in other cultural contexts.

## 5. Conclusions

The Parent–Child Communication Quality Scale, developed in this study, is a second-order measurement model based on the self psychology theory. It consists of four first-order factors, namely mirroring, idealization, empathy, and appropriate response, with a total of 15 items. The theoretical construction of this measurement model was supported by the results of the CFA and this scale exhibited satisfactory reliability and criterion validity. Therefore, the PCCQS can be used as an effective tool to measure children’s perceived parent–child communication quality.

## Figures and Tables

**Table 1:** Items and factor loading of the PCCQS (Father) (*N* = 545).

Content of Items	Four-Factor					One-Factor	
	Factor Loading				Commonality	Factor Loading	Commonality
	Mirroring	Idealization	Empathy	Appropriate Response			
1. I feel very close to my parents in communication.	−0.048	**0.931**	−0.014	0.040	0.831	**0.770**	0.593
2. When I am in a bad mood, my parents can calm me down in time.	0.162	**0.725**	0.020	0.046	0.794	**0.820**	0.673
3. My parents employ a friendly manner when talking with me, which makes me feel secure.	0.035	**0.592**	0.300	0.013	0.749	**0.820**	0.672
4. Communication with my parents brings me a sense of inner warmth.	0.288	**0.453**	0.263	−0.031	0.784	**0.862**	0.744
5. My parents often praise me for my good ideas.	**0.928**	0.235	−0.249	−0.022	0.830	**0.798**	0.637
6. My parents support me in pursuing my good ideas.	**0.787**	0.077	0.069	−0.045	0.754	**0.806**	0.650
7. My parents frequently observe and commend instances of my behavior that they perceive as positive.	**0.738**	0.056	0.102	0.041	0.787	**0.843**	0.711
8. My parents offer me praise when I feel a sense of self-worth and self-esteem.	**0.790**	−0.209	0.219	0.088	0.780	**0.807**	0.651
9. In communication, my parents endeavour to comprehend my perspective.	0.385	−0.131	**0.638**	0.029	0.789	**0.840**	0.705
10. During the chat, my parents can feel my emotions and moods.	0.048	0.067	**0.857**	−0.086	0.787	**0.811**	0.658
11. My parents are very understanding and can often say what I want to say.	−0.063	0.139	**0.804**	0.032	0.775	**0.818**	0.669
12. Parents will listen to me attentively.	−0.089	0.037	**0.869**	0.080	0.779	**0.804**	0.646
13. My parents are capable of responding to my misbehavior in a composed and affectionate manner.	−0.068	0.076	0.006	**0.863**	0.758	**0.691**	0.478
14. My parents can keep calm when I fail to follow their advice.	−0.016	0.068	−0.031	**0.900**	0.828	**0.726**	0.528
15. My parents can offer guidance gently when my behavior is inappropriate.	0.142	−0.080	0.049	**0.805**	0.784	**0.742**	0.550
Eigenvalue	9.565	0.620	0.980	0.646		9.565	
Variance contribution (%)	63.765	4.132	6.533	4.305		63.765	

Notes: PCCQS = Parent–Child Communication Quality Scale. The bold text marks the dimension to which this item belongs and the factor loading for this item.

**Table 2:** Items and factor loading of the PCCQS (Mother) (*N* = 545).

Content of Items	Four-Factor					One-Factor	
	Factor Loading				Commonality	Factor Loading	Commonality
	Mirroring	Idealization	Empathy	Appropriate Response			
1. I feel very close to my parents in communication.	−0.177	**0.915**	0.221	−0.113	0.792	**0.750**	0.562
2. When I am in a bad mood, my parents can calm me down in time.	0.101	**0.701**	0.053	0.061	0.730	**0.799**	0.638
3. My parents employ a friendly manner when talking with me, which makes me feel secure.	0.214	**0.670**	−0.159	0.210	0.756	**0.804**	0.646
4. Communication with my parents brings me a sense of inner warmth.	0.171	**0.634**	0.151	−0.013	0.762	**0.831**	0.690
5. My parents often praise me for my good ideas.	**0.855**	0.226	−0.191	−0.001	0.801	**0.792**	0.627
6. My parents support me in pursuing my good ideas.	**0.623**	0.151	0.215	−0.133	0.683	**0.773**	0.598
7. My parents frequently observe and commend instances of my behavior that they perceive as positive.	**0.804**	0.073	0.030	−0.019	0.754	**0.794**	0.630
8. My parents offer me praise when I feel a sense of self-worth and self-esteem.	**0.857**	−0.265	0.213	0.051	0.761	**0.761**	0.579
9. In communication, my parents endeavour to comprehend my perspective.	0.286	−0.018	**0.584**	0.118	0.778	**0.849**	0.721
10. During the chat, my parents can feel my emotions and moods.	0.009	0.080	**0.817**	−0.003	0.772	**0.798**	0.636
11. My parents are very understanding and can often say what I want to say.	0.094	0.048	**0.714**	0.045	0.720	**0.793**	0.628
12. Parents will listen to me attentively.	−0.063	0.193	**0.767**	0.045	0.799	**0.826**	0.683
13. My parents are capable of responding to my misbehavior in a composed and affectionate manner.	−0.025	−0.092	0.037	**0.922**	0.771	**0.660**	0.435
14. My parents can keep calm when I fail to follow their advice.	0.001	−0.020	0.080	**0.850**	0.794	**0.727**	0.529
15. My parents can offer guidance gently when my behavior is inappropriate.	−0.027	0.155	−0.025	**0.800**	0.753	**0.724**	0.524
Eigenvalue	9.128	1.039	0.655	0.605		9.128	
Variance contribution (%)	60.853	6.930	4.367	4.032		60.853	

Notes: PCCQS = Parent–Child Communication Quality Scale. The bold text marks the dimension to which this item belongs and the factor loading for this item.

**Table 3:** Fit indices for the CFA Models of the PCCQS.

Model		χ^2^	df	χ^2^/df	RMSEA (90%CI)	SRMR	CFI	TLI
Father	One-factor model	623.343	90	6.926	0.117 (0.108–0.125)	0.047	0.899	0.883
	First-order, four-factor model	238.727	84	2.842	0.065 (0.055–0.075)	0.028	0.971	0.963
	Second-order, four-factor model	240.805	86	2.800	0.064 (0.055–0.074)	0.029	0.971	0.964
Mother	One-factor model	506.859	90	5.632	0.103 (0.095–0.112)	0.044	0.906	0.891
	First-order, four-factor model	267.253	84	3.182	0.071 (0.061–0.080)	0.031	0.959	0.949
	Second-order, four-factor model	270.691	86	3.148	0.070 (0.061–0.080)	0.032	0.959	0.949

Notes: PCCQS = Parent–Child Communication Quality Scale; CFI = comparative fit index; TLI = Tucker–Lewis index; SRMR = standardized root mean square residual; RMSEA = root mean square error of approximation.

**Table 4:** Reliability indices of the PCCQS (*N* = 1095).

Item		Cronbach’s α Coefficient	Split-Half Reliability
Father	Mirroring	0.898	0.880
	Idealization	0.907	0.904
	Empathy	0.900	0.902
	Appropriate response	0.859	0.736
	Total score	0.960	0.922
Mother	Mirroring	0.886	0.873
	Idealization	0.886	0.885
	Empathy	0.885	0.878
	Appropriate response	0.839	0.706
	Total score	0.953	0.927

Notes: PCCQS = Parent–Child Communication Quality Scale.

**Table 5:** Correlation analysis between the PCCQS (father) and the PACS (*N* = 1095).

	1	2	3	4	5	6	7	8
1. Idealization	1							
2. Mirroring	0.815 **	1						
3. Empathy	0.831 **	0.812 **	1					
4. Appropriate response	0.680 **	0.671 **	0.722 **	1				
5. PCCQS	0.925 **	0.918 **	0.933 **	0.844 **	1			
6. OFC	0.747 **	0.747 **	0.790 **	0.695 **	0.825 **	1		
7. PFC	0.308 **	0.292 **	0.323 **	0.309 **	0.335 **	0.324 **	1	
8. PACS	0.650 **	0.639 **	0.687 **	0.623 **	0.716 **	0.815 **	0.812 **	1

Notes: ** *p* < 0.01. PCCQS = Parent–Child Communication Quality Scale; PACS = Parent–Adolescent Communication Scale; OFC = Openness in Family Communication; PFC = Problems in Family Communication.

**Table 6:** Correlation analysis between the PCCQS (mother) and the PACS (*N* = 1095).

	1	2	3	4	5	6	7	8
1. Idealization	1							
2. Mirroring	0.799 **	1						
3. Empathy	0.811 **	0.788 **	1					
4. Appropriate response	0.684 **	0.652 **	0.711 **	1				
5. PCCQS	0.914 **	0.908 **	0.926 **	0.846 **	1			
6. OFC	0.735 **	0.696 **	0.784 **	0.677 **	0.804 **	1		
7. PFC	0.314 **	0.271 **	0.288 **	0.292 **	0.319 **	0.309 **	1	
8. PACS	0.636 **	0.586 **	0.652 **	0.594 **	0.680 **	0.790 **	0.827 **	1

Notes: ** *p* < 0.01. PCCQS = Parent–Child Communication Quality Scale; PACS = Parent–Adolescent Communication Scale; OFC = Openness in Family Communication; PFC = Problems in Family Communication.

## Data Availability

The data in this study are available from the corresponding authors upon reasonable request.
